# Editorial: The role of immune response in overnutrition-induced metabolic syndrome

**DOI:** 10.3389/fimmu.2023.1295751

**Published:** 2023-09-22

**Authors:** Xiliang Du, Xinwei Li, Xudong Sun, Wanhai Qin

**Affiliations:** ^1^ State Key Laboratory for Diagnosis and Treatment of Severe Zoonotic Infectious Diseases, Key Laboratory for Zoonosis Research of the Ministry of Education, Institute of Zoonosis, and College of Veterinary Medicine, Jilin University, Changchun, China; ^2^ Heilongjiang Provincial Key Laboratory of Prevention and Control of Bovine Diseases, College of Animal Science and Veterinary Medicine, Heilongjiang Bayi Agricultural University, Daqing, China; ^3^ Department of Physiology and Pharmacology, Snyder Institute for Chronic Diseases, University of Calgary, Calgary, AB, Canada

**Keywords:** overnutrition, metabolic syndrome, immune response, inflammation, diseases

## Introduction

The unhealthy state of metabolic disorder caused by long-term overnutrition is the driving force of metabolic syndrome (Mets) characterized by insulin resistance, hypertension and dyslipidaemia, etc (1). Mets also is considered as a chronic state of low-grade inflammatory state marked by elevated circulating proinflammatory cytokines. The excessive production of proinflammatory cytokines has been linked to an increased risk of obesity, type 2 diabetes, fatty liver, and cardiovascular disease (2, 3). Of note, metabolites from food, dietary ingredients, and natural products can regulate immune response and manipulate inflammatory state. Hence, it is meaningful to identify the mechanisms of overnutrition-induced Mets and explore inflammatory signaling pathways and mediators during the development of Mets, as well as to find potential strategy to treat low-grade chronic inflammation of Mets.

Numerous scholars have directed their attention towards this topic and have acquired interesting discoveries. Within the Research Topic “*The Role of Immune Response in Overnutrition-induced Metabolic Syndrom*e”, four recent studies investigated the underlying mechanism, diagnostic biomarkers and treatment of diseases linked to Mets.

Alzheimer’s disease (AD) has an association with Mets. Clinical and epidemiological evidence indicates that Mets clusters promote the development of AD through various mechanisms. Identifying biomarkers is essential for diagnosing and treating many diseases. However, our understanding of the shared diagnosis and genes associated with both MS and AD is limited. Li et al. obtained eight common diagnostic genes in AD and Mets via the machine learning algorithm. The immune filtration analysis showed that four genes are highly expressed in different immune cell subpopulations. The common mechanism of AD and metabolic syndrome may involve pathways associated with glucose metabolism. Furthermore, the impact of glucose metabolism on AD patients could potentially be mediated by NK cells and B cells. The single-cell sequencing analysis suggested that *SNRPG* may act as a key gene related to glucose metabolism in AD patients. This study provides insights for the diagnosis and treatment of AD and Mets.

Atherosclerosis (AS) is a complex disease with multiple causes. One of the factors contributing to AS is metabolic disorder (Mets). Researchers have investigated the potential therapeutic effects of Trichosanthes kirilowii and Allium macrostemon in managing AS-related diseases. However, the exact underlying mechanism is still not fully understood. Abnormal DNA methylation can also contribute to the development of AS. In a study conducted by Jia et al. a combined approach of MC-seq and RNA-seq was employed to investigate the effects of Gualou-xiebai herb pair (GXHP) treatment on foam cell models induced by ox-LDL treatment in RAW264.7 cells. The results suggested that GXHP appeared to reverse changes in gene expression by modulating abnormal hypermethylation and hypomethylation, resulting in reduced protein levels related to the PI3K-Akt signaling pathway in foam cells. This study has delineated the mechanism of action of GXPH as a novel methylation reagent in AS. Furthermore, it has provided novel insights into the exploration of disease mechanisms mediated by Mets.

Mets is also linked to a higher risk of gout. It was generally believed that the inflammatory response only occurred in the urate deposition stage of gouty arthritis. While the involvement of the NLRP3/IL-1b inflammatory signaling pathway has been confirmed in gout arthritis, its contribution to the overall development of gout remains uncertain. In the work by Wu et al. in the pathological progression of gout, the upregulation of Xanthine oxidase expression facilitates uric acid production, disrupts oxidative stress equilibrium, generates a substantial quantity of reactive oxygen species, triggers the activation of *NLRP3* inflammatory corpuscles, and induces the release of IL-1b in the quail gout model induced by over-nutrition. Meanwhile, they successfully obtained primary synovial fibroblasts in quail, thereby facilitating future investigations. This study holds significant reference value for the research and treatment of gout induced by Mets resulting from overnutrition.

Serotonin has been demonstrated to exacerbate diet-induced obesity, insulin resistance, and non-alcoholic fatty liver disease in mice. It also affects the recruitment and function of white blood cells during inflammation. The study conducted by Hoch et al. elucidated that the absence of serotonin transporter in a knockout mouse model exacerbates obesity related inflammation. The inflammatory response in adipose tissue is attributed to elevated recruitment of leukocytes into obese visceral adipose tissue. This consequently exacerbates dysfunction of adipose tissue, disrupts systemic glucose regulation, and leads to liver steatosis. This study provides valuable information in the investigation of diseases related to metabolic syndrome and the selection of therapeutic medications.

Collectively, these data, ideas and findings described in this series of articles pave the way for further research in the topic.

## Author contributions

XD: Writing – original draft. XL: Writing – review & editing. XS: Writing – review & editing. WQ: Writing – review & editing.
